# Complexity of the *Mycoplasma fermentans* M64 Genome and Metabolic Essentiality and Diversity among Mycoplasmas

**DOI:** 10.1371/journal.pone.0032940

**Published:** 2012-04-03

**Authors:** Hung-Wei Shu, Tze-Tze Liu, Huang-I Chan, Yen-Ming Liu, Keh-Ming Wu, Hung-Yu Shu, Shih-Feng Tsai, Kwang-Jen Hsiao, Wensi S. Hu, Wailap Victor Ng

**Affiliations:** 1 Laboratory Science in Medicine, Department of Biotechnology, Institute of Biotechnology in Medicine, National Yang Ming University, Taipei, Taiwan, Republic of China; 2 Genome Research Center, National Yang Ming University, Taipei, Taiwan, Republic of China; 3 Institute of Biomedical Informatics, National Yang Ming University, Taipei, Taiwan, Republic of China; 4 Institute of Genome Sciences, Department of Life Sciences, National Yang Ming University, Taipei, Taiwan, Republic of China; 5 Division of Molecular and Genome Medicine, National Health Research Institute, Zhunan Town, Miaoli County, Taiwan, Republic of China; 6 Department of Medical Research and Education, Taipei Veterans General Hospital, Taipei, Taiwan, Republic of China; 7 Department of Education and Research, Taipei City Hospital, Taipei, Taiwan, Republic of China; University of Edinburgh, United Kingdom

## Abstract

Recently, the genomes of two *Mycoplasma fermentans* strains, namely M64 and JER, have been completely sequenced. Gross comparison indicated that the genome of M64 is significantly bigger than the other strain and the difference is mainly contributed by the repetitive sequences including seven families of simple and complex transposable elements ranging from 973 to 23,778 bps. Analysis of these repeats resulted in the identification of a new distinct family of Integrative Conjugal Elements of *M. fermentans*, designated as ICEF-III. Using the concept of “reaction connectivity”, the metabolic capabilities in *M. fermentans* manifested by the complete and partial connected biomodules were revealed. A comparison of the reported *M. pulmonis*, *M. arthritidis*, *M. genitalium*, *B. subtilis*, and *E. coli* essential genes and the genes predicted from the M64 genome indicated that more than 73% of the Mycoplasmas essential genes are preserved in *M. fermentans*. Further examination of the highly and partly connected reactions by a novel combinatorial phylogenetic tree, metabolic network, and essential gene analysis indicated that some of the pathways (e.g. purine and pyrimidine metabolisms) with partial connected reactions may be important for the conversions of intermediate metabolites. Taken together, in light of systems and network analyses, the diversity among the *Mycoplasma* species was manifested on the variations of their limited metabolic abilities during evolution.

## Introduction

*Mycoplasma*, a member of the class Mollicutes, is a genus lacking a rigid bacterial cell wall. A number of species of this genus are medically and agriculturally important. Extensive genetic and genomic investigations had been carried out to shed light on their biology and pathogenicity [1], [2], [3], [4], [5], [6], [7], [8], [9], [10]. In addition, Mycoplasmas are genetically simple bacteria with drastically reduced genomes and thus are interesting to study because of the presumably limited but crucial metabolic capabilities and biological activities. To date the genomes of at least twenty species, with sizes ranging from 0.58 (*M. genitalium*) to 1.36 Mbp (*M. penetrans*), have been completely determined [4], [5], [6], [7], [10], [11], [12], [13], [14], [15], [16], [17], [18], [19], [20], [21], [22], [23], [24] (http://www.ncbi.nlm.nih.gov/genomes/lproks.cgi). These sequences contained the basic and crucial information for better understanding of this interesting group of microorganisms.

In spite of intensive basic and clinical research on *M. fermentans* in recent years, its role and involved molecular mechanism in HIV pathogenesis, sexually-transmitted genital tract infection, systemic infection, rheumatic disorders, chronic fatigue syndrome and other diseases has remained elusive [25], [26], [27], [28], [29], [30], [31], [32], [33], [34], [35]. *M. fermentans* is a fastidious microorganism isolated or detected commonly from human genitourinary and respiratory tracts [36], [37] and correlated with diseases in both healthy individuals and AIDS patients. It was first described several decades before its detection in patients with AIDS in the late 1980s and was considered an opportunistic pathogen or a sexually transmissible cofactor contributing to the pathology and pathogenesis of HIV-associated diseases [38]. The detection of this species in the peripheral blood lymphocytes and urine of AIDS patients in a previous study suggested that it might have the ability to act as polyclonal activators of both T and B lymphocytes to stimulate the replication of HIV. It is thought to behave as a cofactor or immunomodulator in HIV-related diseases [39]. *M. fermentans* might be a systemic pathogen, causing fatal disease owing to the infection of the bone marrow in non-HIV-infected patients [40]. It may actively invade cultured cells such as HeLa cells and survive as an intracellular pathogen [41]. They had also been found to be present intracellularly or adhered to cell surface [42]. On the other hand, *M. fermentans* may play a critical role in genital tract infection and rheumatic disorders such as rheumatoid arthritis [43], [44]. In addition, several studies have been conducted to elucidate the relationship of *Mycoplasma* and chronic fatigue syndrome (CFS) [45]. These studies showed *Mycoplasma* species including *M. fermentans*, *M. hominis*, and *M. penetrans* could be detected in the patients with CFS, however no solid evidence that these organisms act as a cause of CFS has been reported. In conclusion, *M. fermentans* is a clinically interesting *Mycoplasma* species and recent studies had brought the attention towards its possible involvement in several critical human diseases.

Three types of transposable elements including insertion sequences (IS), ICEFs (integrative conjugal element of *M. fermentans*), and prophage had been found in some *M. fermentans* strains [46], [47], . These elements had a wide range of size distribution ranging from 973 to 23,778 bp and some were present as multiple copies. For instance, nine copies of IS*1630*, which encoded an IS*30* family like transposase and had diverse target site specificity, have been detected in *M. fermentans* [47]. IS*1550*, also known as ISMi *1*, had been demonstrated to be a possibly active element in *E. coli*. In addition, IS*1550* had been found to be integrated into IS*1630* and be inserted by the large transposable element ICEF [46], [47], [48], [49]. ICEF is similar to conjugative self-transmissible integrating elements (constins), but due to the absence of the homologs of integrases, transposases or recombinases, it is considered to be distinct from typical constins in the mechanism of integration-excision. Moreover, the approximately 16-kb *M. fermentans* prophage ΦMFV1 might be integrated as single or multiple copies in the genome [49]. Taken together, the presence of these elements might be important for the plasticity of the genome and evolution of *M. fermentans* [49], [50], [51].

To increase our understandings of the biology of *M. fermentans*, which might be helpful for revealing its pathogenic roles in the suspected diseases, a comprehensive comparative genomic analysis was performed on *M. fermentans* strains M64 and JER and the other Mycoplasmas. We examined the DNA sequences which contributed to the dramatic difference in size between the M64 and JER genomes. The metabolic abilities of *M. fermentans* M64 were analyzed by a systems analysis method which was based on the evaluation reaction connectivity. Finally, an integrated analysis of a phylogenetic tree, metabolic network, and essential genes was carried out to uncover the essentiality and diversity of metabolic reactions in *M. fermentans* M64 during evolution.

## Results and Discussion

### M. fermentans M64 Harbors a Large Number of Transposable Elements

**Figure 1:**
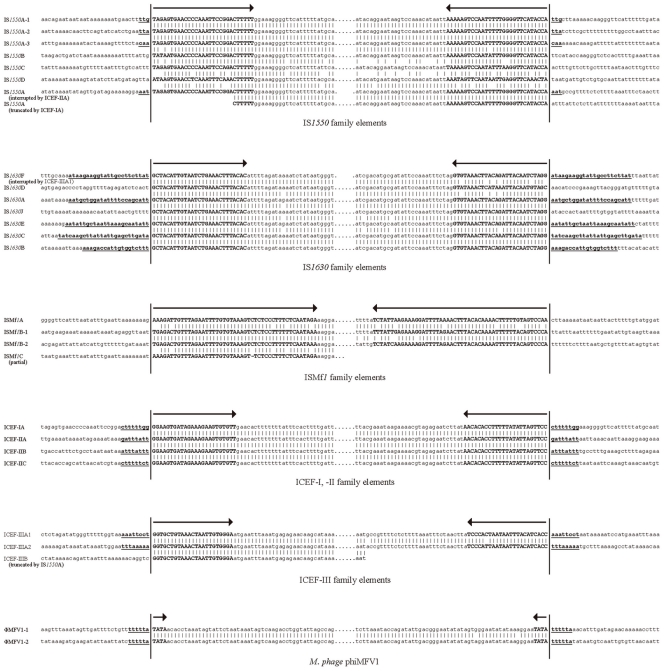
Terminal and junction sequences of the IS elements, ICEFs, and ΦMFV1 prophage DNAs in the M. fermentans M64 genome. The terminal inverted repeats and flanking sequences of 15 complete and 4 partial copies of three families of IS elements including the IS*1550*, IS*1630*, and ISMf*1*, 7 copies of three families of ICEFs, and 2 prophage genomes are shown. Bold uppercase letters with arrows on top indicate the terminal inverted repeats. The target site duplications are shown in underlined bold lowercase. The elements interrupted by other transposable elements are indicated.

Similar to *Mycoplasma mycoides* subsp. *mycoides* [16], sequence analyses indicated that the *M. fermentans* M64 genome also possessed a high density of transposable elements (Figures S1 and S2) [52]. Nine copies of two types of large repetitive sequences and many copies of relative small insertion sequence (IS) elements accounted for 21.6% of the genome (Table 1 and Figure 1) [46], [47], [48], [49]. Among the large repeats were three families of ICEF (22.3 to 23.8 kb) which included four copies of the previously sequenced ICEF-1A (23.8 kb) and the partly characterized ICEF-IIA, B, and C (22.3 kb), and two complete (20.2 and 20.8 kb) and one partial (20.7 kb; 52 bp truncation at the 3′-end) copies of ICEF-III (ICEF-IIIA and B) belonging to a new family discovered in our recent study [52]. Two copies (20.2 and 20.8 kb) of ICEF-III were complete, whereas another one was truncated by IS*1550*A. In addition, there were two copies of ΦMFV1 prophage DNAs (15.8 and 15.6 kb) inserted in the chromosome. A total of 15 complete and 4 incomplete copies of three families of IS elements including eight IS*1550* (973 to 1,416 bp; 6 complete copies, one copy interrupted by ICEF-IIA, and another copy might have been truncated by the transposition of ICEF-IA), seven IS*1630* (1,178 to 1,384 bp; one interrupted by ICEF-IIIA), and four ISMf*1* (1,395 to 1,570 bp; three complete and one incomplete elements) were identified.

**Table 1:** General features of M. fermentans M64 genome.

	Repetitive sequences included*	Repetitive sequences excluded*
Genome size (bp)	1,118,751	887,179
GC content (%)	26.9	27
Gene density (rRNA and, tRNA genes included) (%)	89.7	90.6
Total number of CDSs	1050	820
Number of CDSs with annotated function	576 (54.9%)	507 (61.8%)
Number of hypothetical proteins	332 (31.6%)	210 (25.6%)
Number of conserved hypothetical proteins	142 (13.5%)	103 (12.6%)
Average protein length (a.a.)	314	321
Start codon usage		
AUG	963 (91.7%)	752 (91.7%)
UUG	55 (5.2%)	43 (5.2%)
GUG	32 (3.1%)	25 (3.1%)
Total number of rRNA genes	5	3*
Total number of tRNA genes	35	35
Repetitive transposable elements		
IS elements	19 (4 incomplete)	-
ICEFs	7	-
Prophage, ΦMFV1	2	-
Total length of repetitive sequences (bp)	241,287	-
Repetitive sequences (%)	21.6	-

* Repetitive sequences included the transposable elements and the duplicated 16 and 23S rRNA.

### M. fermentans strains M64 and JER had Significant Difference in Large Transposable Elements

**Figure 2:**
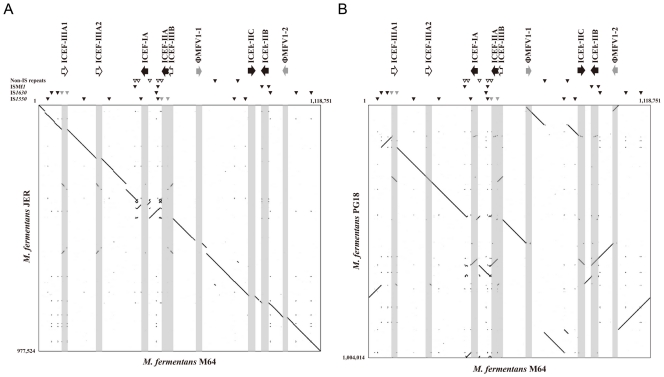
Dotplot of the genomes of M. fermentans strains M64 versus JER (A) and PG18 (B). The graphs were generated with the *Gepard* 1.21 with word length of 20 and window size of 0 [78]. The long repetitive sequences are indicated by horizontal arrows: ICEF-I and -II (unfilled arrows), ICEF-III (black arrows), and ΦMFV1 (gray arrows). The locations of the IS elements of the ISMf*1*, IS*1550* and IS*1630* families are indicated by small black and gray (elements interrupted by ICEFs) triangles. The two types of non-IS small duplications are indicated by small filled (16–23S rRNA operon) and open triangles (uncharacterized repetitive sequence containing a hypothetical protein-coding gene).

Sequence comparison indicated that the genome of *M. fermentans* M64 (1,118,751 bp) [52] is 141 and 115 kb larger than that of the JER strain (977,524 bp) [21] and the nearly complete PG18 strain (1,004,014 bp; Accession no. AP009608) [53]. The differences are mainly contributed by the differences between the copy numbers of ICEF and ΦMFV1 prophage DNAs in these genomes (Figure 2). Notably, there are still gaps of unknown size and potential assembly problems in the PG18 genome, thus the actual difference between it and M64 genome remains to be accurately demonstrated. The *M. fermentans* JER genome essentially lacked all of the previously identified ICEF elements and ΦMFV1 prophage DNA but contained two partial copies of the newly identified ICEF-III elements [48], [49]. Regardless of these differences, the organization of the M64 and JER genome sequence were largely similar. On the other hand, at least two apparently complete ICEFs and one ΦMFV1 prophage DNA were found within the almost complete PG18 genome. Nonetheless, expectably, the organization of the PG18 genome is considerably different from that of the M64 possibly due to the gaps and sequence arrangement problems in an incomplete genome.

Analyses of the integrity and flanking sequences of the IS elements and ICEFs suggested that the current *M. fermentans* M64 genome architecture at least in part might result from the transposition and/or recombination events of the elements in different periods of time. Almost all of the elements, except for members of IS*1550*A-1, -2 and -3 subfamilies, are polymorphic in DNA sequences. As shown in Figure 1, the four ICEFs (ICEF-I, -IIA, -IIB, and -IIC) are terminated with 24-bp imperfect inverted repeats and flanked by distinct 8-bp target site duplications. The new ICEF-IIIs, except for the incomplete ICEF-IIIB which is disrupted by IS*1550*A, are all terminated with 23-bp imperfect inverted repeats and flanked by 8-bp target site duplications. Sequence comparison of ICEF-IA, IIA, and IIIA and phylogenetic analysis of 11 intact ICEs (Integrative Conjugal Elements) in six *Mycoplasma* species indicated that ICEF-III is distinct from the other two ICEF families in *M. fermentans* and unique in all reported ICEs (Figure S3). Both copies of ΦMFV1 prophage genomes are terminated with 4-bp inverted repeats and flanked by identical 6-bp (TTTTTA) target site duplications, suggesting that this could be the preferred transposition target sequence, as reported previously [49]. The seven complete IS*1550* are terminated with 29-bp imperfect inverted repeats and four of them are flanked by 3-bp direct repeats. The seven complete IS*1630* are terminated with 27-bp heterogeneous inverted repeats and four of these elements are flanked by 16-, 23-, 25-, and 26-bp duplicated junction sequences. The three complete copies of ISMf*1* contain 47-bp imperfect terminal inverted repeat but none of them have noticeable target site duplication. The absence of target site duplication in some of these elements suggested that post-transposition DNA rearrangements or additional transposition events might have occurred prior to strain evolution since those families of transposable elements, except for ΦMFV1 prophage, are present in *M. fermentans* strains M64, JER, and PG18. The high frequency of DNA rearrangements in *M. fermentans* has been shown by Hu *et al*. in a previous study [51].

### Pan-genome Analysis Across the M64, JER, and PG18 Strains

**Figure 3:**
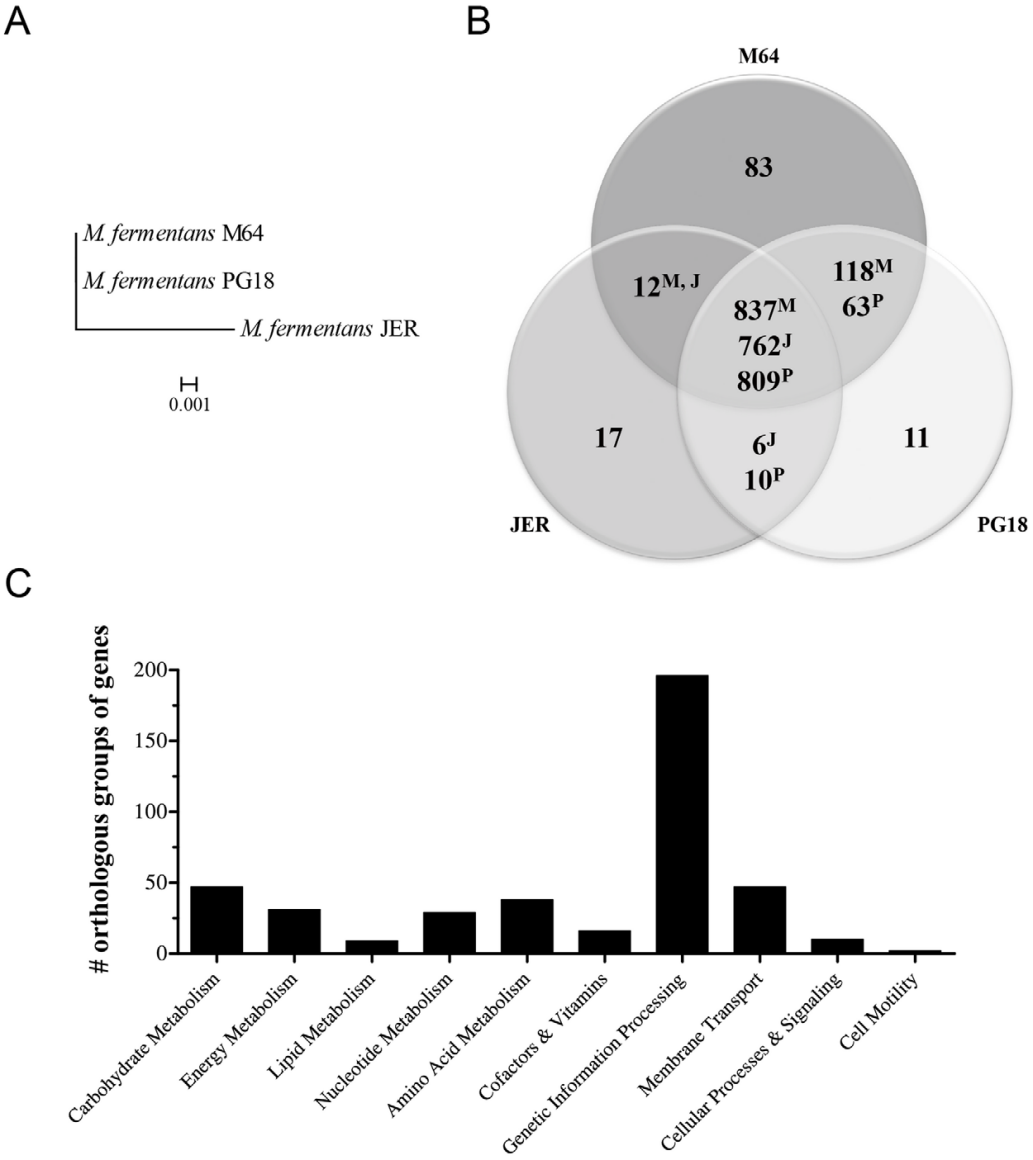
Overview of the pan-genome analysis across the M. fermentans strains M64, JER, and PG18. **A.** The phylogenetic tree of the *M. fermentans* strains M64, JER, and PG18. The concatenated sequences of the genes *dnaE*, *pyk*, and *rpoA* shared by the three strains of *M. fermentans* were used for phylogenetic analysis. The maximum-likelihood method was performed to reconstruct this tree using *PHYML* 3.0. The scale bar stands for the estimated number of nucleotide substitutions per site. **B.** The pan-genome of the *M. fermentans* strains M64, JER, and PG18. Venn diagram exhibits the number (including duplications) of genes unique to each strain or shared by these strains (*BLASTP* E-value <10^−5^). Superscripts represent the name initials of the strains which show the indicated numbers of genes with homologies to other strains. The numbers of genes in the nearly complete PG18 genome may be slightly underestimated due to the presence of gaps and potential unresolved duplications in genome assembly. **C.** Functional distribution of 378 orthologous groups of genes in the core genome. Some of the proteins have more than one KEGG pathway/biomodule annotation.

To unveil the variations in gene content among *M. fermentans* M64, JER, and PG18, sequence similarity searches across all predicted proteins were carried out by using *BLASTP* to dissect the gene repertoire in these strains. The homologs were recognized under the criterion of E-value <10^−5^. Due to differential activities of the events of gene duplication and/or horizontal gene transfer, the number of genes shared by any two or present in all three strains might be different. For instance, the gene *MfeM64YM_0098* of M64 had a total of 8 homologs (*MfeM64YM_0098*, *MfeM64YM_0108*, *MfeM64YM_0229*, *MfeM64YM_0238*, *MfeM64YM_0385*, *MfeM64YM_0474*, *MfeM64YM_0483*, and *MfeM64YM_0992*), whereas PG18 and JER, respectively, had only 4 (*MBIO_0117*, *MBIO_0267*, *MBIO_0276*, and *MBIO_0750*) and 5 (*MFE_08000*, *MFE_02760*, *MFE_02860*, *MFE_04903*, and *MFE_04960*) gene homologs. Thus, pan-genome analysis exhibited 762∼837 genes constituting the core genome of *M. fermentans* strains M64 (837), JER (762), and PG18 (809) (Figure 3B). In agreement with the phylogenetic relationship among these three strains (Figure 3A), more gene homologs are shared by M64 (955) and PG18 (872) than M64 (849) and JER (774), corroborating the closer relationship between M64 and PG18 obtained from the analysis of the concatenated sequence of *dnaE*, *pyk*, and *rpoA* (Figure 3B, Table S1). Expectably, M64 with the largest chromosome rich in mobile elements possesses much more strain specific genes (83 genes) than JER (17) and PG18 (11). Further sequence analysis indicated that most strain specific genes encoded proteins without annotated function. Based on the annotations in the KEGG Orthology database, the gene pool in the core genome (762∼837 genes) contained 378 well-annotated orthologous groups of genes with approximately half belonging to the genetic information processing biomodules (Figure 3C).

### M. fermentans M64 Metabolic Capabilities Revealed by Reaction Connectivity-Based Analyses

Global analyses of *M. fermentans* M64 metabolism indicated only 131 of the 1,050 predicted proteins mapped to the metabolic pathways in KEGG database (KEGG Mapper; http://www.genome.jp/kegg/tool/map_pathway1.html). Apparently, it had a relatively large number of proteins in carbohydrate (57 proteins) and nucleotide (31 proteins) metabolic networks, and a scattered distribution of proteins in amino acid (25 proteins), cofactors and vitamin (18 proteins), and lipid (8 proteins) metabolic networks (Figure S4). A gross examination of the reactions with predicted proteins in the global metabolic network immediately perceived that *M. fermentans* could only synthesize or degrade a limited number of the metabolites as established for other *Mycoplasma* species.

Evaluation of the functionality of the 58 pathways with M64 proteins via the examination of connectivity of reactions [54] found only nine pathways contained five or more connected reactions (Table 2). Several carbohydrate metabolic pathways seemed to have sufficient connected reactions to drive the synthesis or degradation of the pertinent metabolites. The presence of a complete glycolysis/gluconeogenesis pathway (18 connected reactions) suggested that *M. fermentans* M64 is capable of catabolizing D-glucose to pyruvate using the Embden-Meyerhoff-Parnas (EMP) pathway. The pyruvate may then be converted to acetyl-CoA, acetate, or D-lactate with the pyruvate metabolism pathway which contained a single cluster of nine connected reactions driven by ten predicted proteins. Due to the lack of citrate cycle enzymes, it may use D-glucose by fermentative degradation. On the other hand, the pentose phosphate pathway, which contained a large cluster of 13 and a small cluster of two connected reactions, may be utilized to synthesize phosphoribosyl diphosphate (PRPP) that may then enter the purine (11 connected reactions) and pyrimidine (10 reactions) metabolism pathways for nucleotide synthesis. Although only 6 genes were mapped to the fructose and mannose metabolism pathway, these encoded proteins form a cluster of six connected reactions to drive the conversion of D-fructose to glyceraldehyde-3-phosphate, which may then enter the glycolysis pathway for further degradation.

**Table 2:** Summary of the number of M. fermentans M64 predicted genes, reactions, connected reactions in KEGG metabolic pathways.

Metabolism	KEGG Pathway	# Genes	# Reactions	Connectivity in Reactions
Amino Acid	Arginine and proline metabolism	5	5	3,1,1
	Cysteine and methionine metabolism	2	2	2
Carbohydrate	Glycolysis/Gluconeogenesis	15	18	17,1
	Pentose phosphate pathway	12	15	13,2
	Pyruvate metabolism	9	8	8
	Amino sugar and nucleotide sugar metabolism	10	11	7,2,2
	Fructose and mannose metabolism	5	6	6
	Galactose metabolism	9	7	2,1,1,1,1,1
	Pentose and glucuronate interconversions	6	6	2,1,1,1,1
	Starch and sucrose metabolism	4	5	2,1,1,1
	Propanoate metabolism	2	2	2
Cofactors and Vitamins	Nicotinate and nicotinamide metabolism	5	6	5,1
	Pantothenate and CoA biosynthesis	3	3	3
	One carbon pool by folate	3	4	4
	Thiamine metabolism	2	2	2
	Riboflavin metabolism	1	2	2
	Lipoic acid metabolism	1	2	2
Energy	Oxidative phosphorylation	9	2	2
Lipid	Glycerophospholipid metabolism	6	5	5
	Glycerolipid metabolism	3	2	2
Nucleotide	Pyrimidine metabolism	22	22	8,3,2,2,2,1,1(×4)
	Purine metabolism	20	27	6,6,3,3,3,2,2,1,1
Other Amino Acids	Selenoamino acid metabolism	3	5	2,1,1,1

* The number of reactions within each “group of linked reactions” and “orphan reaction” is separated by commas.

Neither the purine nor the pyrimidine pathways for *de novo* nucleotides synthesis seemed to be complete and thus *M. fermentans* M64 must rely on external supply of nucleotides or nucleotide synthesis intermediates to produce the building blocks of DNA and RNA (Table 2 and Figure S5). Apparently, *M. fermentans* M64 possesses the enzymes to form subnetworks of connected reactions between the RNA synthesis and the metabolism of (**i**) guanosine, guanine, GMP, GDP, and GTP, (**ii**) adenosine, adenine, AMP, ADP, and ATP, and (**iii**) UTP and CTP; and between DNA synthesis and the metabolisms of (**iv**) guanosine, guanine, deoxyguanosine, dGMP, dGDP, and dGTP, and (**v**) dAMP, dADP, and dATP.

*M. fermentans* M64, which is similar to the other *Mycoplasma* species, had very few amino acid metabolism proteins predicted from the genome. Only two of the amino acid metabolic pathways had connected reactions – the “arginine and praline” and “cysteine and methionine” pathways had clusters of 3 and 2, respectively, reactions. The presence of arginine deiminase (ArcA), ornithine carbamoyltransferase (ArcB), and carbamate kinase (ArcC) formed a cluster of reactions catalyzing the degradation of arginine to ammonia in the “arginine and proline metabolism” is in agreement with the previous study in which *M. fermentans* was shown to be capable of utilizing arginine (62). Notwithstanding some of the proteins did not form highly connected reaction clusters, they might still be important for the survival of the microorganism. For instance, the proline iminopeptidase (Pip) may release proline from peptide and the aspartate-ammonia ligase (AsnA) catalyzes the interconversion of aspartate and asparagine. In addition, S-adenosylmethionine synthetase (MetK) is an indispensable enzyme for the conversion of methionine to S-adenosyl-L-methionine, which can then be used as the substrate of cytosine-specific methyltransferase (Dcm) for DNA, and rRNA methylation. Therefore, the overall network and connectivity analysis suggested that *M. fermentans* M64 must rely on external supply of all amino acids for protein synthesis.

### Conservation of Genes and Biomodules among the Mycoplasmas

**Figure 4:**
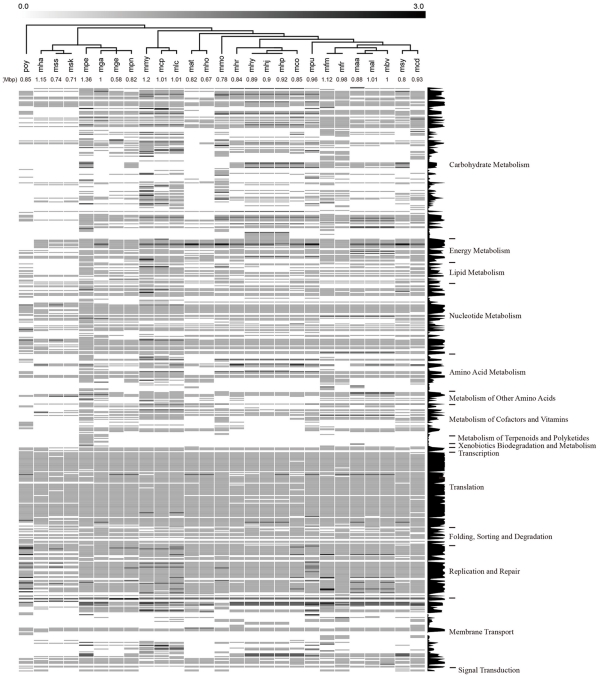
Clustering analysis of the metabolic and cellular process proteins of 27 Mycoplasma species/strains and Phytoplasma OY. The order of the *Mycoplasma* species is according to their phylogenetic relationship (23S rRNA tree). Each column represents the proteins of one single species/strain and each row (horizontal line) represents the copy number (grey color scale on top) of one gene. The sizes of the genomes in Mbp are indicated below the 3-letter abbreviated names of the organisms. Functional clustering and comparison of the proteins among species were based on KO (KEGG Orthology) assignments. The conservation of each gene among the *Mycoplasma* species (i.e. the number of *Mycoplasma* species possessing the gene) is shown on the right side of clustering result. *Abbreviations* – mha: *M. haemofelis*; mss: *M. suis* Illinois; msk: *M. suis* KI3806; mpe: *M. penetrans*; mga: *M. gallisepticum*; mge: *M. genitalium*; mpn: *M. pneumoniae*; mmy: *M. mycoides*; mcp: *M. capricolum*; mlc: *M. leachii*; mat: *M. arthritidis*; mho: *M. hominis*; mmo: *M. mobile*; mhr: *M. hyorhinis*; mhy: *M. hyopneumoniae* 232; mhj: *M. hyopneumoniae* J; mhp: *M. hyopneumoniae* 7448; mco: *M. conjunctivae*; mpu: *M. pulmonis*; mfm: *M. fermentans* M64; mfr: *M. fermentans* JER; maa: *M. agalactiae* PG2; mal: *M. agalactiae* 5632; mbv: *M. bovis* PG45; msy: *M. synoviae*; mcd: *M. crocodyli*; and poy: *Phytoplasma* OY.

Clustering analysis of the proteins from 27 sequenced *Mycoplasma* and *Phytoplasma* species indicated that most genes responsible for DNA replication, nucleotide excision, homologous recombination, transcription, and translation and a significant fraction of the membrane transporters including some of the ABC transporters and members of protein transport and bacterial secretion system are highly conserved among these species (Figure 4). The genes related to carbohydrate metabolism are conserved in almost all species except for *M. haemofelis*, *M. suis*, *M. arthritidis*, and *M. hominis*. Several genes accounting for pyruvate metabolism and other carbohydrate metabolisms were lost in them, such as *pdhA*, *pdhB*, *pdhC*, and *pdhD*. Moreover, distinct patterns of other metabolisms could also be observed for *M. haemofelis* and *M. suis.* Intriguingly, *M. haemofelis* and *M. suis* are hemotropic Mycoplasmas. They belong to a special group of Mycoplasmas, also known as hemoplasma, with a tropism for red blood cells [55], [56]. This distinctive propensity may be associated with the evolution of the pool of metabolism- and cellular process-related genes. *M. arthritidis* and *M. hominis*, whose hosts are rodent and human respectively, also form a unique phylogenetic clade, referred to as the *M. hominis* cluster, in Mycoplasmas. They both cannot carry out the glucose fermentation but capable of arginine hydrolysis [57], [58]. All of the *Mycoplasma* species exhibited conserved energy metabolism-related genes including genes encoding NADH dehydrogenase and F-type ATPase aside from cytochrome-containing complex, indicating a truncated electron transport chain. Thus, the ATP generation proceeds via inefficient substrate-level phosphorylation in the flavin-terminated respiratory pathway rather than oxidative phosphorylation [59], [60], [61]. In addition, the pentose phosphate pathway, an alternative to glycolysis, may generate NADPH for reductive biosynthesis reactions in *Mycoplasma* species. The presence of only a limited number of recognizable genes encoding the proteins in the biomodules for metabolisms of most carbohydrates, amino acids, and cofactors and vitamins are conserved, suggesting that most, if not all, of these metabolic pathways are defective unless there are cryptic genes which can carry out the missed function [62]. This observation is in agreement with the requirements of a wide spectrum of substrates and factors for the growth of *Mycoplasma*.

The analysis of the proteins involved in genetic information processing unambiguously indicated that they are widely distributed among the members of the sequenced *Mycoplasmataceae* family (Figure 4). Interestingly, the nucleotide metabolism and genetic information processing deservedly exhibited highly conserved profiles of involved genes, whereas *M. haemofelis* and *M. suis* seemed to be deficient in some genes. The DNA polymerase III holoenzyme of *M. fermentans* and the other *Mycoplasma* species resembles the Gram (+) type and is simpler than that of the Gram (-) model organism [63]. The *Mycoplasma* species polymerase III holoenzymes are made up of the same subunits as that of the Gram (+) *Bacillus subtilis* which comprises the α, β, γ, τ, δ, and δ′ subunits. Although the gene encoding the ε subunit with 3′–5′ exonuclease activity could not be found in their genomes, the structure of the Mycoplasmas α subunit protein resembles the PolC-type (Gram (+) type) α subunit, which contains both the DnaE (α subunit) and DnaQ (ε subunit) domains with the DNA polymerase and 3′–5′ exonuclease, respectively, activities suggesting a *B. subtilis*-like mechanism of DNA replication.

The DNA-directed RNA polymerase α, β, and β′ subunits are sufficient to form a minimal core (ββ′α2) of RNA polymerase for catalyzing the polymerization of nucleoside triphosphates into RNA [64]. The RNA polymerase primary σ factor, which promotes the attachment of RNA polymerase to a specific site in the promoter for transcription initiation, is highly conserved among the sequenced *Mycoplasma* species. In addition to these subunits, the δ subunit which is ubiquitous among the Gram (+) bacteria were also found to be conserved in some of the *Mycoplasma* species including *M. haemofelis*, *M. suis*, *M. leachii*, *M. capricolum*, *M. mycoides*, *M. penetrans*, *M. pneumoniae*, *M. genitalium*, and *M. gallisepticum* but not in *M. fermentans*. Although one gene (MfeM64YM_0186) encoding a protein containing the domain of RNA polymerase δ subunit (pfam05066) is present on the chromosome of *M. fermentans* M64, the E-value is only 6.3×10^−5^ and its protein size (89 a.a.) is markedly smaller than those (134∼170 a.a.) of other sequenced Mycoplasmas. Importantly, the sequence similarity analysis of the protein MfeM64YM_0186 demonstrated that all significant hits (E-value<10^−5^) were annotated as putative uncharacterized protein. Therefore, MfeM64YM_0186 was considered a hypothetical protein and might be degenerated from the typical RNA polymerase δ subunit. The δ subunit is involved in both the initiation and recycling phases of transcription, and may also serve as a virulence factor [65]. The specificity of transcription and the efficiency of RNA synthesis are increased in the presence of the δ subunit because of enhanced recycling. Previous studies reported that mutation of the δ subunit gene would result in extended lag phase growth [66] and a defect in starvation-induced stationary-phase survival or recovery [67]. This subunit does not exist in all *Mycoplasma* species but is likely responsible for the complex regulation and flexibility of gene expression in those *Mycoplasma* species bearing the δ subunit genes.

The aminoacy-tRNA synthetases and ribosomal genes also have a high degree of conservation among the sequenced Mycoplasmas. Similar to the Gram (+) *B. subtilis*, Mycoplasmas do not possess glutaminyl-tRNA synthetase. They may take a similar strategy as *B. subtilis* and other organisms which lack this enzyme by charging glutamate to both tRNA^Glu^ and tRNA^Gln^ with a nondiscriminating glutamyl-tRNA synthetase and then converting glutamyl-tRNA^Gln^ to glutaminyl-tRNA^Gln^ with glutamyl-tRNA^Gln^ amidotransferase (GatABC complex). This is partly supported by observation that *M. fermentans* and the other 21 *Mycoplasma* species and strains have a protein homolog (MfeM64YM_0687 in *M. fermentans*) more similar to the nondiscriminating glutamyl-tRNA synthetase of *B. subtilis* (61.7% similarity) than the discriminating *E. coli* enzyme (53% similarity). *Mycoplasma* species has slightly less ribosomal subunit proteins than those of *E. coli* (total 55). Furthermore, the genes encoding the GatABC complex are also present in *M. fermentans* M64 and other sequenced Mycoplasmas. Upon the manually curated annotations from KEGG database (http://www.genome.jp/kegg/pathway/ko/ko03010.html) and validations for smaller proteins (<100 a.a.) by performing *TBLASTN*, a total of 47 proteins of the ribosome complex are highly conserved among the sequenced *Mycoplasma* species and strains. The S1, S6, S21, L9, and L32 subunits are absent in some of the species and S22, L25, and L30 subunits could not be found in any of the species. In line with our observations, Kawauchi et al. [68] employed two-dimensional polyacrylamide gel electrophoresis to analyze the *M. capricolum* ribosomes, and identified 30 proteins of large ribosomal subunit and 21 proteins of small ribosomal subunit. The *M. capricolum* ribosome lacks S1, S22, L25, and L30 proteins. S1 protein is present in seven species belonging to three close phylogenetic clusters including the *M. lipophilum*, *M. synoviae*, and *M. pulmonis* clusters [58] and two other species. Furthermore, S21 protein restrictively exists in nine species of the hemoplasma, pneumoniae, and spiroplasma groups [57]. Together, these observations suggested that some of these proteins may show the specificity for certain phylogenetic clades and be dispensable in Mycoplasmas as reported previously [12], [69].

### Potential Essential Genes in M. Fermentans

**Figure 5:**
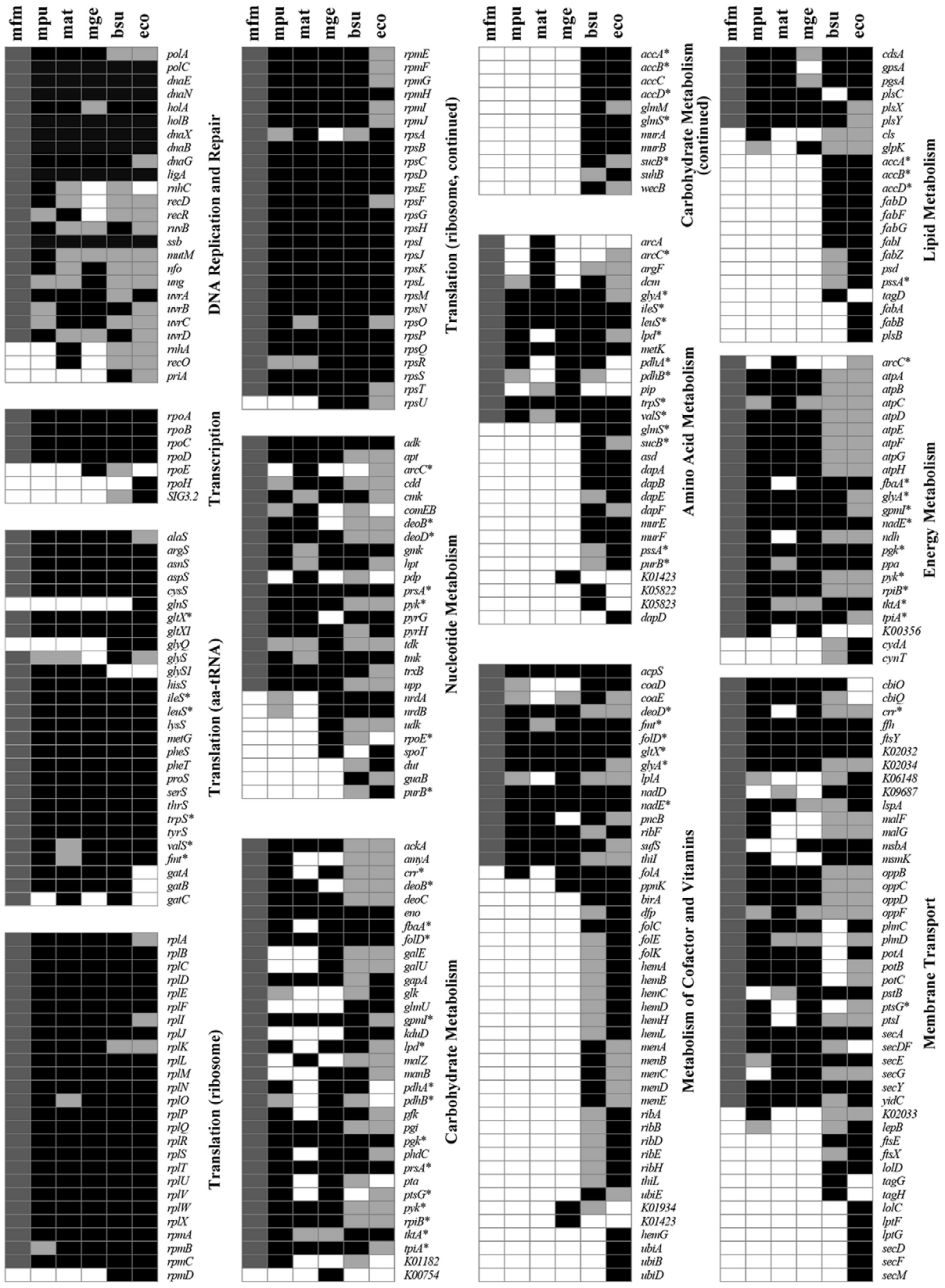
Comparison of predicted genes of M. fermentans M64 with the reported essential genes of five bacteria species. *BLASTP* was implemented to compare the proteome of *M. fermentans* M64 (mfm) against the proteomes of *M. pulmonis* (mpu), *M. arthritidis* (mat), *M. genitalium* (mge), *B. subtilis* (bsu), and *E. coli* (eco). The gene names (or KEGG Orthology numbers) and KEGG functional categories are indicated. Black rectangles indicate the essential genes in mpu, mat, mge, bsu, and eco. Light grey rectangles indicate the genes which had not been shown to be essential. Dark gray rectangles indicate the potential essential genes in *M. fermentans*. White rectangles indicate the missing genes. The genes involved in more than one functional category are indicated by asterisk.

The essential genes from the closely related *M. pulmonis*, *M. arthritidis*, and *M. genitalium* and the distantly related *B. subtilis* and *E. coli* identified in previous studies were compared with the proteins predicted from the *M. fermentans* M64 genome to shed light on *M. fermentans* likely essential genes necessary for a minimal self-replicating cell (Figure 5) [12], [69], [70], [71], [72]. It is notable that more essential genes in *M. pulmonis*, *M. arthritidis*, and *M. genitalium* than the other two species have homologs in *M. fermentans*. Approximately 303 (97.7%) of the 310 *M. pulmonis*, 315 (75.7%) of the 416 *M. genitalium*, 282 (73.8%) of the 382 *M. genitalium*, 150 (55.3%) of the 271 *B. subtilis*, and 168 (55.4%) of the 303 *E. coli* essential gene homologs were found in the *M. fermentans* M64 genome. Although the matched essential genes in *M. fermentans* have yet to be validated, a subset of essential genes in *Mycoplasma* species that seem to be distinct from the two distantly related bacteria were identified. The higher similarity of *M. fermentans* with *M. pulmonis* than with *M. arthritidis* and *M. genitalium* is in accordance with their phylogenetic distance (Figure S6). A relatively higher degree of conservation is observed for the essential genes participating in translation and transcription. In addition, a significant fraction of the essential genes involved in replication and repair, nucleotide metabolism, amino acid metabolism, and membrane transport also appeared to be highly conserved in the analyzed species. On the other hand, some of the proteins only seemed to be essential to one or more but not all species. It is conceivable that the genome reduction was accompanied by a shift of the essential gene sets during the evolution as shown in carbohydrate metabolism and membrane transport (Figure 5).

### Conservation of Residual Metabolism and Essential Genes among the Mycoplasmas

**Figure 6:**
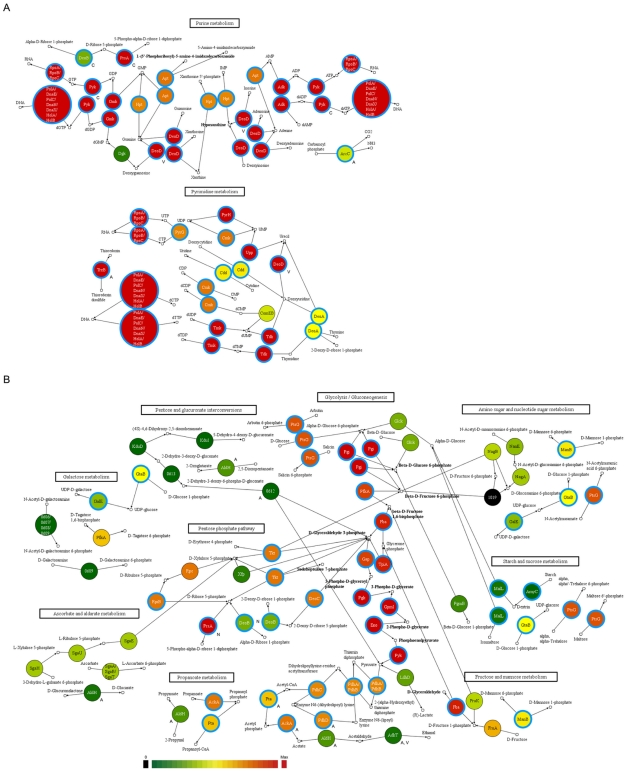
Phylogenetic conservation analysis of nucleotide (A) and carbohydrate (B) metabolic networks of M. fermentans M64 and 20 other Mycoplasma species. *M. fermentans* M64 metabolic networks were served as the references in the comparisons. The small open squares represent the compounds and the colored circles represent the proteins (enzymes) participating in the metabolisms. Circles with thick and light blue circumference denote the potential essential genes inferred from the comparison with *M. pulmonis*, *M. arthritidis*, and *M. genitalium* (Figure 5). The arrows and lines linking the enzymes and substrates indicate the direction of the reactions. Enzymes involved in more than one metabolism are annotated with a single letter code of the other metabolisms (A: Amino acids; C: Carbohydrate; N: Nucleotide; V: cofactors and Vitamins) next to the circles. The color scale (bottom) indicates the phylogenetic conservation of proteins in the analyzed *Mycoplasma* species.

Analysis of the conservation of the genes among the Mycoplasmas indicated that some of the residual or partial metabolic capabilities in *M. fermentans* are highly conserved among the *Mycoplasma* species. A cross examination of the essential genes in *M. pulmonis* [69], *M. arthritidis* [12], and *M. genitalium* [70] suggested that many of the conserved genes in the residual metabolic functions are likely essential in Mycoplasmas. The incomplete nucleotide and carbohydrate metabolism were chosen as the examples to illustrate this property. Notwithstanding Mycoplasmas lack the full capability of *de novo* nucleotide synthesis, a total of 21 and 22 genes in purine and pyrimidine metabolic pathways, respectively, were found in the *M. fermentans* genome. Phylogenetic comparison of the nucleotide metabolic networks indicated that the genes present in *M. fermentans* are more conserved in purine metabolism than those in the pyrimidine pathway among the 21 sequenced *Mycoplasma* species (Figure 6A). The enzymes which catalyzed clusters of connected reactions in purine metabolic pathway, except for Dgk and DeoB only present in six and twelve other species, respectively, are highly conserved among the Mycoplasmas. Most species have the enzymes for the synthesis of ATP from adenine or adenosine, and GTP and dGTP from guanine or guanosine. However, three phylogenetically related species - *M. fermentans*, *M. agalactiae*, and *M. bovis* in the *M. lipophilum* cluster and two other related species - *M. arthritidis* and *M. hominis* in the *M. hominis* cluster [58] lack the ribonucleotide diphosphate reductase complex for the conversion of ADP to dADP and thus dATP might be synthesized from dADP or dAMP or uptake from an extracellular source. On the other hand, the enzymes in the pyrimidine metabolic pathway are relatively less conserved among the Mycoplasmas. Due to the lack of at least one key enzyme, all sequenced species may rely on external supply of UTP, dCTP, and dTTP from the hosts or culture media. For instance, *M. fermentans* is short of genes encoding dCTP deaminase involved in the conversion of CTP to UTP and nucleoside-diphosphate kinase responsible for the conversion of UDP to UTP, dCDP to dCTP, and dTDP to dTTP. Moreover, ribonucleoside-triphosphate reductase catalyzing the conversion of CTP to dCTP is also absent in it. Some of the species such as *M. fermentans* carry the *pyrG* gene and thus may be able to convert UTP to CTP.

Of the carbohydrate metabolism, *M. fermentans* M64 and the other *Mycoplasma* species seemed to possess a highly conserved glycolysis pathway as well as the incomplete and partially conserved pentose phosphate and pyruvate metabolic networks (Figure 6B). Upstream of glycolysis pathway, α-D-glucose and β-D-glucose may enter the pathway through the enzyme GlcK which was only found in *M. fermentans* M64 and seven other species. The joint presence of AmyC and MalL in a few species including *M. fermentans*, *M. pulmonis*, *M. hyorhinis*, and *M. crocodyli* suggested that starch may be converted to dextran and then to α-D-glucose and subsequently enter the glycolysis pathway in these species. In addition, the presence of the FruA, FruK, and Fba, which have different degree of conservation, suggested that fructose may also be utilized by *M. fermentans* M64 and some other *Mycoplasma* species. In contrast to the highly conserved Fba which interconverts D-fructose 1,6-bisphosphate and glycerone phosphate plus D-glyceraldehyde 3-phosphate, FruA does not exist in all of two hemoplasma species and six species in the *M. hominis* group including *M. lipophilum*, *M. hominis*, and *M. synovia*e clusters. FruK is present in all of three species belonging to the spiroplasma group, two species in the pneumoniae group, and two species in the hominis group including M. hyorhinis and M. fermentans. Within the pentose phosphate pathway, the enzymes Tkt, Rpe, and RpiB for the conversion of D-glyceraldehyde 3-phosphate to D-ribulose 5-phosphate are conserved in all species except for the hemoplasma. The enzyme PrsA, which converts D-ribulose 5-phosphate to 5-Phospho-alpha-D-ribose 1-diphosphate (PRPP) could be found in all *Mycoplasma* species. Many *Mycoplasma* species seemed to be able to catabolize pyruvate into acetyl-CoA and acetate through a conserved pathway, whereas only some have a complete set of enzymes to further metabolize pyruvate to lactate or ethanol. *M. fermentans*, *M. agalactiae*, *M. bovis*, and *M. crocodyli* in the hominis group, *M. mycoides* and *M. leachii* in the spiroplasma group, and *M. penetrans* have lactate dehydrogenase catalyzing the interconversion between lactate and pyruvate. Of these species, *M. fermentans*, *M. crocodyli*, and *M. penetrans* are also capable of converting acetate to ethanol by exerting aldehyde dehydrogenase and alcohol dehydrogenase which only exist in a couple of species. This implies that *M. fermentans* M64 and several other *Mycoplasma* species may be able to undergo lactic or alcoholic fermentation. Similarly, when the enzymes in the incomplete amino acid and vitamin and cofactor metabolic networks were examined, some of the enzymes also found to be conserved, suggesting that at least some of the residual metabolic activities may be related to their survival (figure S7).

### Genes Outside of the Mycoplasma Clade

Sequence analysis identified 15 genes in *M. fermentans* M64 with no homolog among all of significant *BLASTP* hits coming from *Mycoplasma* species, suggesting that they are not conserved in the *Mycoplasma* clade (See comment on the Table S2). These genes might be the vestiges of the genome reduction from the last common Gram-positive ancestor, or be the horizontally transferred genes. The genes were dispersedly distributed on the chromosome. Among these, 7 genes were closest to the Gram-positive bacteria, and 8 genes were closest to Gram-negative bacteria. All of 7 Gram-positive bacteria belonged to Firmicutes, of which 6 belonged to Clostridia. Those 7 genes with highest homologies to Firmicutes were regarded as the possible preserved genes after the onset of the evolution of genome minimization. In addition, the 8 genes closest to Gram-negative bacteria, including 4 species belonging to Bacteroidetes/Chlorobi group, 3 to Cyanobacteria, and 1 to Spirochaetes, might have been transferred laterally to *M. fermentans* M64. No UGA codon was detected in these 8 putative horizontally transferred gene candidates, suggesting that these genes might not have been transferred for a long period and thus was insufficient to induce the codon bias. The proteins encoded by the 15 genes consisted of 5 conserved hypothetical proteins, 3 AAA+superfamily ATPases, and 7 different proteins. During the evolution of *M. fermentans* M64, AAA+superfamily ATPase might play an important role in the compensation for genome minimization because this protein is accountable for a large variety of diverse functions via molecular remodeling dependent of the energy from ATP hydrolysis, such as protein degradation, DNA recombination, replication, repair, and so on [73]. Moreover, the *hsdS* among the 15 genes encodes a subunit of type I restriction enzyme which could protect the hosts against foreign DNA such as those from bacteriophages. For those 15 gene candidates, the protein phylogeny was reconstructed for *M. fermentans* M64 homolog and the twelve most similar homologs of different organisms in the *BLASTP* results, and further, *M. fermentans* M64 and those twelve organisms were analyzed for the 23S rRNA phylogeny to elucidate their evolutionary relationship. Regarding the ORF *MfeM64YM_1027*, *M. fermentans* M64 occupied a distinct, individual branch with strong branch support and was distant from *Clostridium perfringens* in the 23S rRNA phylogenetic tree (Figure S8A). However, *M. fermentans* M64 MfeM64YM_1027 was closest to the homolog of *C. perfringens*, raising the possibility that this gene might be a remnant after genome minimization (Figure S8B). Similar result was also observed for another gene (MfeM64YM_0060) which product was closest to the homolog of a Gram-negative bacterium (Figure S8C and D). Taken together, the preserved genes after the onset of genome reduction and horizontally transferred gene candidates appeared to play some critical roles in the versatility of a small genome and shaping of a limited metabolism.

### Conclusion

The genome of *M. fermentans* M64, despite the interference of a large proportion of repetitive sequences when assembling the whole genome shotgun sequences, has been successfully determined in our previous study [52]. Rare conservation of transposable elements among the Mycoplasmas suggests that most elements were integrated in the genomes after speciation. Clustering analysis of *Mycoplasma* genes indicated the conservations of degenerated metabolisms and cellular processes, suggesting that some of the remaining metabolic functions might be indispensable. On account of the lack of complete amino acid, nucleotide, and vitamin and cofactor synthesis pathways, these compounds or their intermediate metabolites must be obtained from external sources and entered the cells in the forms such as peptides, amino acids, and nucleotides with poorly understood transport systems. The completion of the M64 and JER strains of *M. fermentans* and the other *Mycoplasma* genomes would facilitate the revelation of these systems and the biology of this interesting group of microorganisms with reduced genomes.

## Materials and Methods

### Comparative Metabolomics

The metabolism of *M. fermentans* M64 and the other *Mycoplasma* species were compared by clustering analysis of the homologous genes. The clustering analysis and comparison of the presence and absence of genes in different functional groups was based on the KO (KEGG Orthology) assignments of the homologous proteins in KEGG database. *M. fermentans* M64 protein sequences were submitted to the KEGG Automatic Annotation Server (KAAS) [74] to obtain the KO number and pathway assignments. While similar information of the other *Mycoplasma* species and *Phytoplasma* OY were extracted from the organism-specific files in the KEGG database.

### Conservation of Metabolisms

A new method for evaluating the phylogenetic conservation of metabolism in a genus/group of organisms was developed to predict the most conserved metabolism among the *Mycoplasma* species. This analysis is based on the hypothesis that the probability of the preservation of an enzymatic reaction in two closely related species should be different from that of two distantly related species. The *M. fermentans* M64 metabolic networks constructed with the KO number and KEGG pathway information served as the reference networks in this analysis. The conservation of enzymes, reactions, and metabolic pathway between *M. fermentans* M64 and the 19 *Mycoplasma* species and *Phytoplasma* OY were calculated with the following equations:
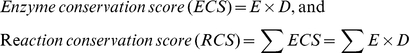
in which, ECS calculates the conservation of the enzyme of the compared species; E is the presence (1) or absence (0) of the enzyme; D is the “Phylogenetic Distance” between *M. fermentans* and the compared species which is expressed as the substitution rate (D) indicated in the 23S rRNA phylogenetic tree of the 21 *Mycoplasma* species and one outgroup species. The phylogenetic tree was constructed with *PHYML* 3.0 [75] and evaluated with the *aLRT* method [76]. The phylogenetic distance was considered as a weight for the enzyme conservation in this analysis. If an enzyme exists in two distantly related species, the enzyme likely has higher conservation in this genus/group of organisms, and *vice versa*. Each RCS represents the conservation of the enzyme orthologs in all of compared species which catalyze the same reaction.

### Essential Genes

The essential genes of *M. pulmonis*, *M. arthritidis*, *M. genitalium*, *B. subtilis*, and *E. coli* identified in previous studies [12], [69], [70], [71], [72] and the other proteins predicted from the genomes were submitted to KAAS to obtain their KO numbers via sequence homology searches. The *M. fermentans* M64 essential genes were predicted by clustering analysis with the essential and putative non-essential proteins according to the KO number and through *BLASTP* [77] analysis of each protein against a consolidated database of the proteomes of the six analyzed species.

### Genes Outside of the Mycoplasma Clade

The *M. fermentans* M64 genes outside of the *Mycoplasma* clade were identified by examining all of significant hits of the *BLASTP* [77] search results against the proteomes of sequenced prokaryotic organisms retrieved from the UniProt database (http://www.uniprot.org/downloads). Genes were considered to be outside of the *Mycoplasma* clade if no hit among all significant matches belonged to *Mycoplasma* species as described previously [15]. Then the closest organisms for genes outside of the *Mycoplasma* clade were assigned as the top non-*Mycoplasma* hit which had more than 70% sequence coverage in the alignment with *M. fermentans* sequence.

### Genome Sequence Accession Numbers

The accession numbers of the *M. fermentans* M64 genome sequence is NC_014921. The species for comparative analyses included *M. fermentans* strain JER (NC_014552), *M. haemofelis* (NC_014970), *M. suis* Illinois (NC_015155), *M. suis* KI3806 (NC_015153), *M. penetrans* (NC_004432), *M. gallisepticum* (NC_004829), *M. genitalium* (NC_000908), *M. pneumoniae* (NC_000912), *M. mycoides* (NC_005364), *M. capricolum* (NC_007633), *M. leachii* (NC_014751), *M. arthritidis* (NC_011025), *M. hominis* (NC_013511), *M. mobile* (NC_006908), *M. hyorhinis* (NC_014448), *M. hyopneumoniae* 232 (NC_006360), *M. hyopneumoniae* J (NC_007295), *M. hyopneumoniae* 7448 (NC_007332), *M. conjunctivae* (NC_012806), *M. pulmonis* (NC_002771), *M. agalactiae* PG2 (NC_009497), *M. agalactiae* 5632 (NC_013948), *M. bovis* PG45 (NC_014760), *M. synoviae* (NC_007294), *M. crocodyli* (NC_014014), *Phytoplasma* OY (NC_005303), *B. subtilis* subsp. *subtilis* str. 168 (NC_000964), and *E. coli* str. K-12 substr. MG1655 (NC_000913).

## Supporting Information

Figure S1 **The circular representation of the M. fermentans M64 genome.** The ticks on the outermost concentric circle indicate the relative genomic positions in base pairs, where position one is the first base of the upstream intergenic region of *dnaA* gene. Second and third concentric circles: predicted protein coding genes on the plus and minus strands, respectively. The various functional groups of the predicted genes (color coded) are categorized according to the COG classification. Fourth concentric circle: locations of rRNA (gray) and tRNAs (green) genes. Fifth concentric circle: ICEF-I and IIs (red arrows), ICEF-III (green arrows), ΦMFV1 prophage (blue arrows), IS elements [IS*1550* (dark brown), IS*1630* (brown), ISMf*1* (orange)], 16S-23S rRNA operons (blue), and non-IS repeats (yellow). Sixth and seventh concentric circles: GC content and GC skew calculated in 5,000 bp of sliding window with 100 bp of step window, respectively.
(TIF)

Figure S2 **Validation of the large repeats and genome assembly of M. fermentans M64.** A. and C. EtBr-stained gel images of restriction enzymes (*BglI*, *MluI*, *KpnI*, and *AdhI*)-digested M64 genomic DNA. These fragments were resolved by CHEF gel electrophoresis in 1% agarose and 0.5X TBE at 6.0 v/cm for approximately 21.5 hr with pulsing times of 0.92 to 19.89 s and 0.92 to 37.34 s, respectively. **B**. and **D**. Southern blot analyses of DNA fragments in **A** and **C**, respectively. The BglI and MluI, KpnI, and AhdI-digested fragments were hybridized with the ICEF-I and ICEF-II (926 bp), ICEF-III (848 bp), prophage ΦMFV1 (920 bp) specific probes, respectively. The Southern blotting was conducted with DIG High Prime DNA Labeling and Detection Starter Kit (Roche, Basel, Switzerland). Lane M: MidRange I PFG Marker (New England Biolabs, Ipswich, Massachusetts); Lane U: Uncut genomic DNA; The size of the marker bands are indicated on the right.
(TIF)

Figure S3 **Comparison among ICEF-IA, ICEF-IIA, and ICEF-IIIA of M. fermentans M64 indicated ICEF-III is a new family of ICE.** **A**., **B**., and **C**. Results of sequence comparison of ICEF-IIIA and ICEF-IA, ICEF-IA and ICEF-IIA, and ICEF-IIA and ICEF-IIIA, respectively. The sequence similarities between ICEF-IIIA and ICEF-IA, ICEF-IA and ICEF-IIA, and ICEF-IIA and ICEF-IIIA are, 45.6%, 91.9%, and 45.4%, respectively. The GC content (sliding window: 120 bp) of each element was plotted on top or bottom of each panel. The arrows indicate the ORFs and the four-digit numbers represent the locus name (i.e., the number follow “MfeM64YM_” in locus tag). The two strips in dark gray stand for the forward and reverse strands of DNA. The direct and complementary matched regions between 2 elements are linked by blue and red lines, respectively. **D**. Phylogenetic tree of the 11 intact Integrative Conjugal Element (ICE) of Mycoplasmas. ICEB, ICEA, ICEF, ICEH, ICEM, and ICEC represent the elements in M. *bovis, M. agalactiae, M. fermentans, M. hyopneumoniae, M. mycoides, and M. capricolum* genomes, respectively. The scale bar stands for the estimated number of nucleotide substitutions per site.
(TIF)

Figure S4 **A global view of M. fermentans metabolic networks showing the connected reactions in carbohydrate (green), nucleotide (blue), amino acids (red), cofactors and vitamins (orange), and lipid (pink) metabolisms.** The map is produced by matching the M64 predicted proteins to metabolic pathways with the “color pathway” tool in KEGG (http://www.genome.jp/kegg/tool/color_pathway.html).
(TIF)

Figure S5 **M. fermentans M64 purine (A) and pyrimidine (B) metabolic pathways contain many small clusters of connected reactions.** Small circles represent the metabolic intermediates and green rectangles with enzyme catalog numbers denote the proteins in *M. fermentans.*
(TIF)

Figure S6 **Phylogenetic tree of the 21 Mycoplasma species analyzed in** **Figure 4** **.** Maximum-likelihood method was performed on the 23S rRNA to reconstruct this tree using *PHYML* 3.0. The branch reliability was evaluated by implementing the aLRT method. *Phytoplasma* OY was set as the outgroup. The scale bar stands for the estimated number of nucleotide substitutions per site.
(TIF)

Figure S7 **Phylogenetic comparative analysis of cofactors and vitamins (A) and amino acid (B) metabolic networks of M. fermentans M64 and 20 other Mycoplasma species.** The small open squares represent the compounds and the colored circles represent the proteins (enzymes) participated in the metabolism. Circles with thick and light blue circumference represent the predicted essential genes. The arrows and lines linking the enzymes and substrates indicate the direction of the reactions. Small rectangle in **B** represents a putative horizontally transferred gene candidate. Enzymes involved in more than one metabolism are indicated with a one letter code (A: Amino acids metabolism; C: Carbohydrate metabolism; N: Nucleotide metabolism; V: metabolism and cofactors and Vitamins) next to the circles. The color scale (bottom) indicates the phylogenetic conservation of the proteins in 21 *Mycoplasma* species.
(TIF)

Figure S8 **Phylogenetic relationships of MfeM64YM_1027 and MfeM64YM_0060 outside of the Mycoplasma clade.** **A**. and **C**. The 23S rRNA trees of the species shown in B and D, respectively. **B**. and **D**. The phylogenetic trees of MfeM64YM_1027 and MfeM64YM_0060, respectively, and their closest homologs present in 12 other species. Maximum-likelihood method was used to reconstruct the trees. The branch reliability was evaluated by implementing the aLRT method. The scale bar stands for the estimated number of nucleotide (**A** and **C**) or amino acid (**B** and **D**) substitutions per site.
(TIF)

Table S1 **Unique and common genes of the M. fermentans strains M64, JER, and PG18.**
(XLSX)

Table S2 **Genes of M. fermentans M64 outside of the Mycoplasma clade.**
(XLSX)
