# Supplementary figures and images for: Complexity of the Mycoplasma fermentans M64 Genome and Metabolic Essentiality and Diversity among Mycoplasmas
Source: PLoS One. 2012 Apr 3;7(4):e32940. doi: 10.1371/journal.pone.0032940 (PMC3317919; doi:10.1371/journal.pone.0032940)

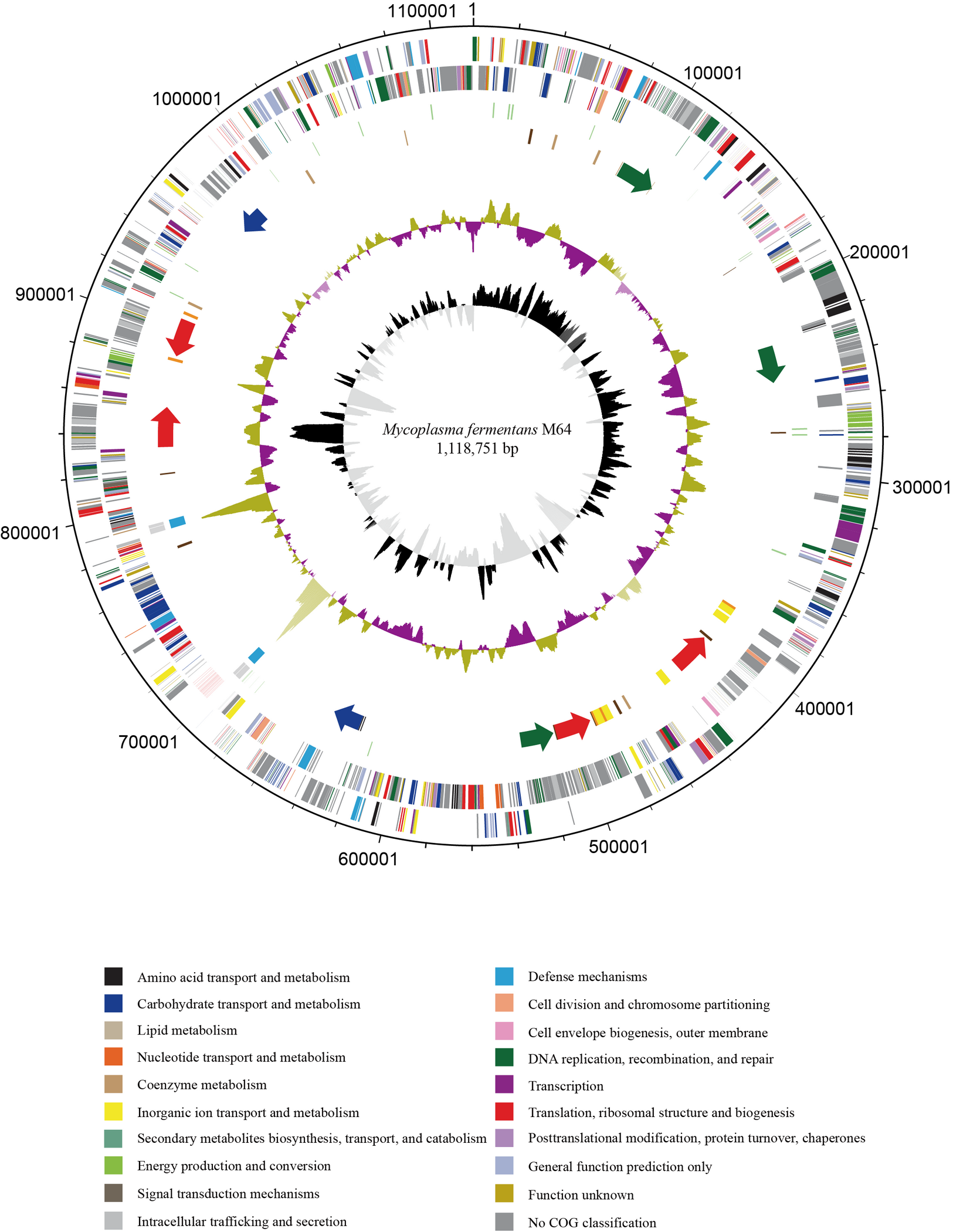

Supplement: Figure S1 — The circular representation of the M. fermentans M64 genome. The ticks on the outermost concentric circle indicate the relative genomic positions in base pairs, where position one is the first base of the upstream intergenic region of dnaA gene. Second and third concentric circles: predicted protein coding genes on the plus and minus strands, respectively. The various functional groups of the predicted genes (color coded) are categorized according to the COG classification. Fourth concentric circle: locations of rRNA (gray) and tRNAs (green) genes. Fifth concentric circle: ICEF-I and IIs (red arrows), ICEF-III (green arrows), ΦMFV1 prophage (blue arrows), IS elements [IS1550 (dark brown), IS1630 (brown), ISMf1 (orange)], 16S-23S rRNA operons (blue), and non-IS repeats (yellow). Sixth and seventh concentric circles: GC content and GC skew calculated in 5,000 bp of sliding window with 100 bp of step window, respectively. (TIF) [file pone.0032940.s001.tif]

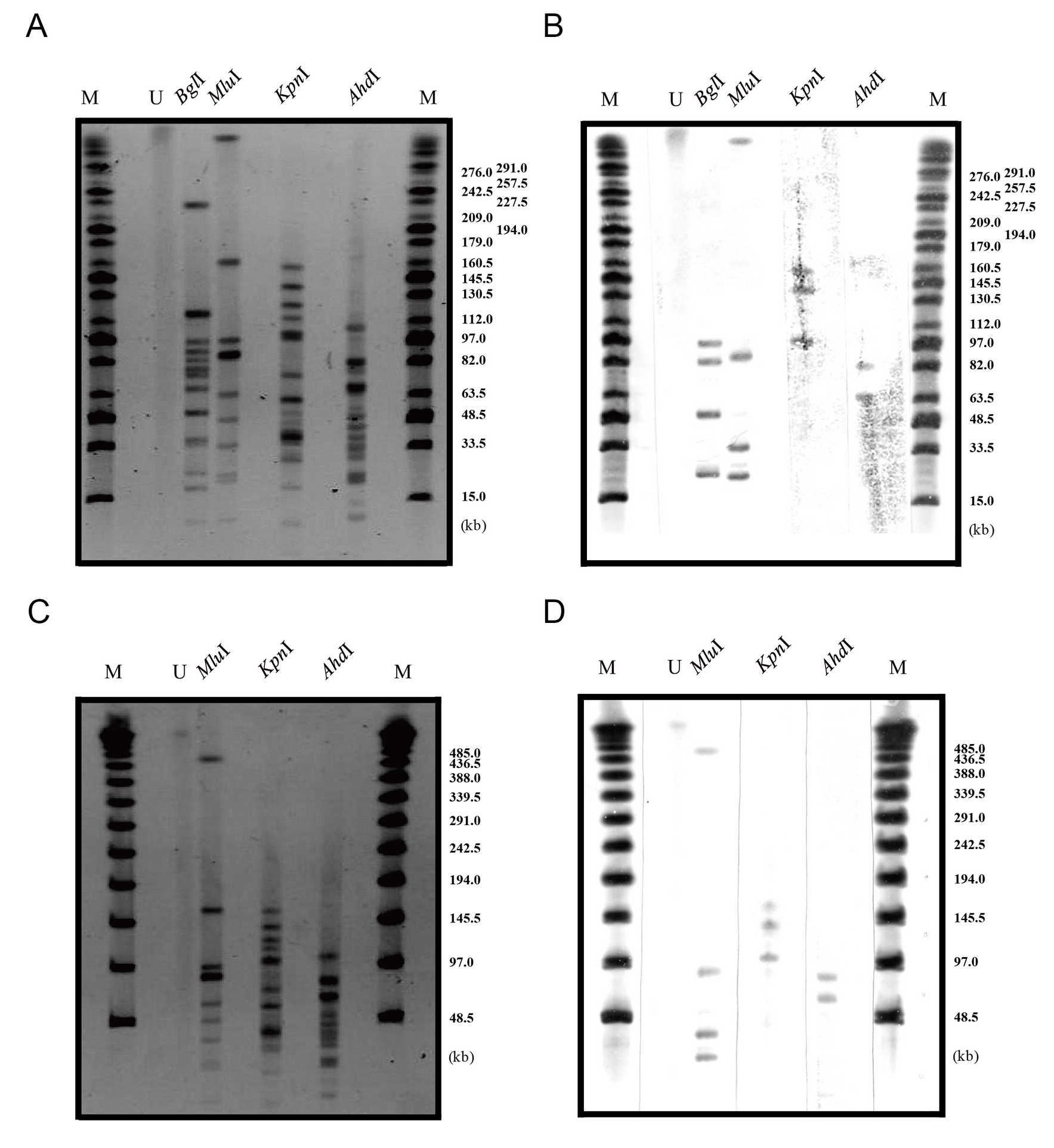

Supplement: Figure S2 — Validation of the large repeats and genome assembly of M. fermentans M64. A. and C. EtBr-stained gel images of restriction enzymes (BglI, MluI, KpnI, and AdhI)-digested M64 genomic DNA. These fragments were resolved by CHEF gel electrophoresis in 1% agarose and 0.5X TBE at 6.0 v/cm for approximately 21.5 hr with pulsing times of 0.92 to 19.89 s and 0.92 to 37.34 s, respectively. B. and D. Southern blot analyses of DNA fragments in A and C, respectively. The BglI and MluI, KpnI, and AhdI-digested fragments were hybridized with the ICEF-I and ICEF-II (926 bp), ICEF-III (848 bp), prophage ΦMFV1 (920 bp) specific probes, respectively. The Southern blotting was conducted with DIG High Prime DNA Labeling and Detection Starter Kit (Roche, Basel, Switzerland). Lane M: MidRange I PFG Marker (New England Biolabs, Ipswich, Massachusetts); Lane U: Uncut genomic DNA; The size of the marker bands are indicated on the right. (TIF) [file pone.0032940.s002.tif]

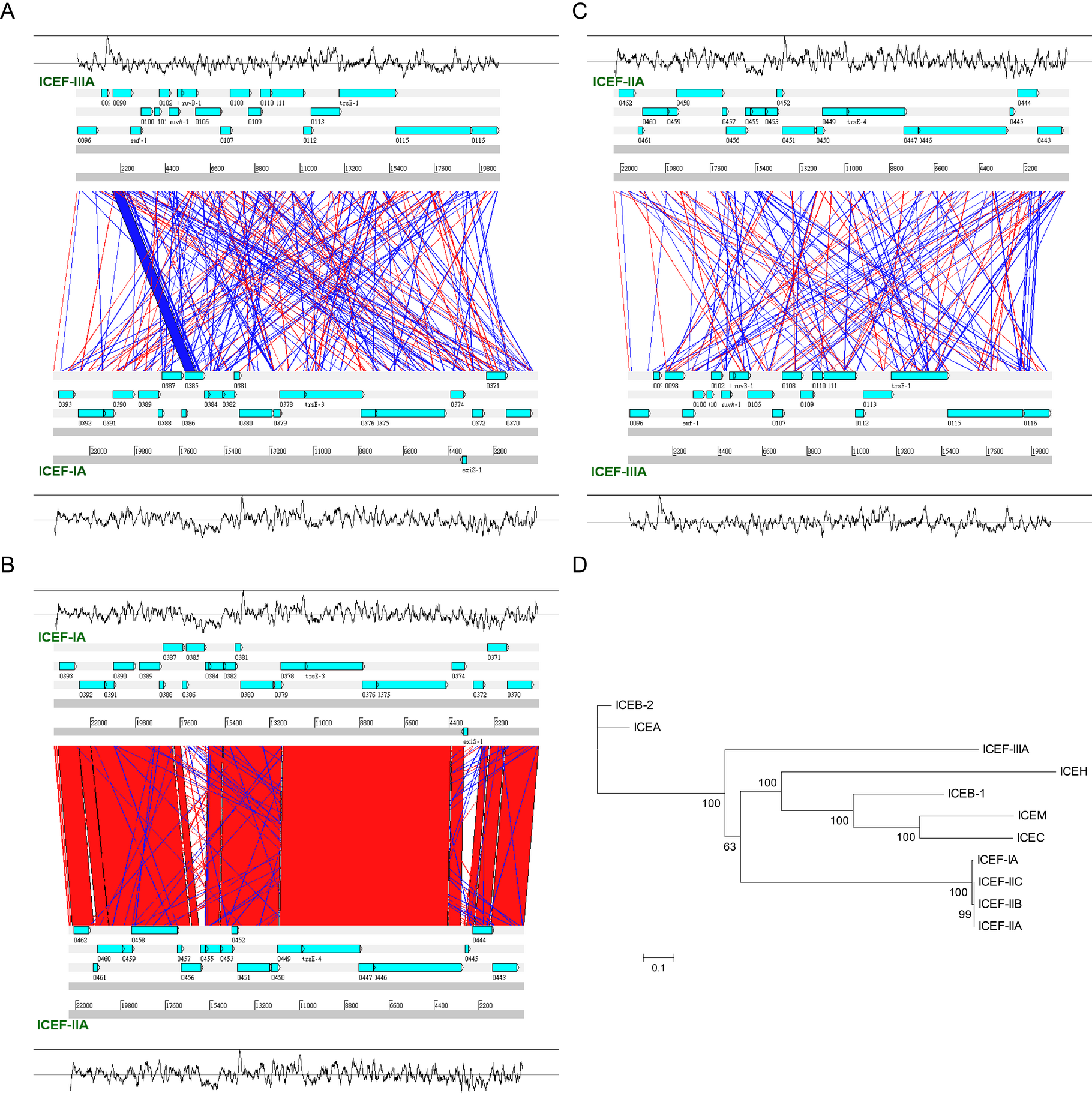

Supplement: Figure S3 — Comparison among ICEF-IA, ICEF-IIA, and ICEF-IIIA of M. fermentans M64 indicated ICEF-III is a new family of ICE. A., B., and C. Results of sequence comparison of ICEF-IIIA and ICEF-IA, ICEF-IA and ICEF-IIA, and ICEF-IIA and ICEF-IIIA, respectively. The sequence similarities between ICEF-IIIA and ICEF-IA, ICEF-IA and ICEF-IIA, and ICEF-IIA and ICEF-IIIA are, 45.6%, 91.9%, and 45.4%, respectively. The GC content (sliding window: 120 bp) of each element was plotted on top or bottom of each panel. The arrows indicate the ORFs and the four-digit numbers represent the locus name (i.e., the number follow “MfeM64YM_” in locus tag). The two strips in dark gray stand for the forward and reverse strands of DNA. The direct and complementary matched regions between 2 elements are linked by blue and red lines, respectively. D. Phylogenetic tree of the 11 intact Integrative Conjugal Element (ICE) of Mycoplasmas. ICEB, ICEA, ICEF, ICEH, ICEM, and ICEC represent the elements in M. bovis, M. agalactiae, M. fermentans, M. hyopneumoniae, M. mycoides, and M. capricolum genomes, respectively. The scale bar stands for the estimated number of nucleotide substitutions per site. (TIF) [file pone.0032940.s003.tif]

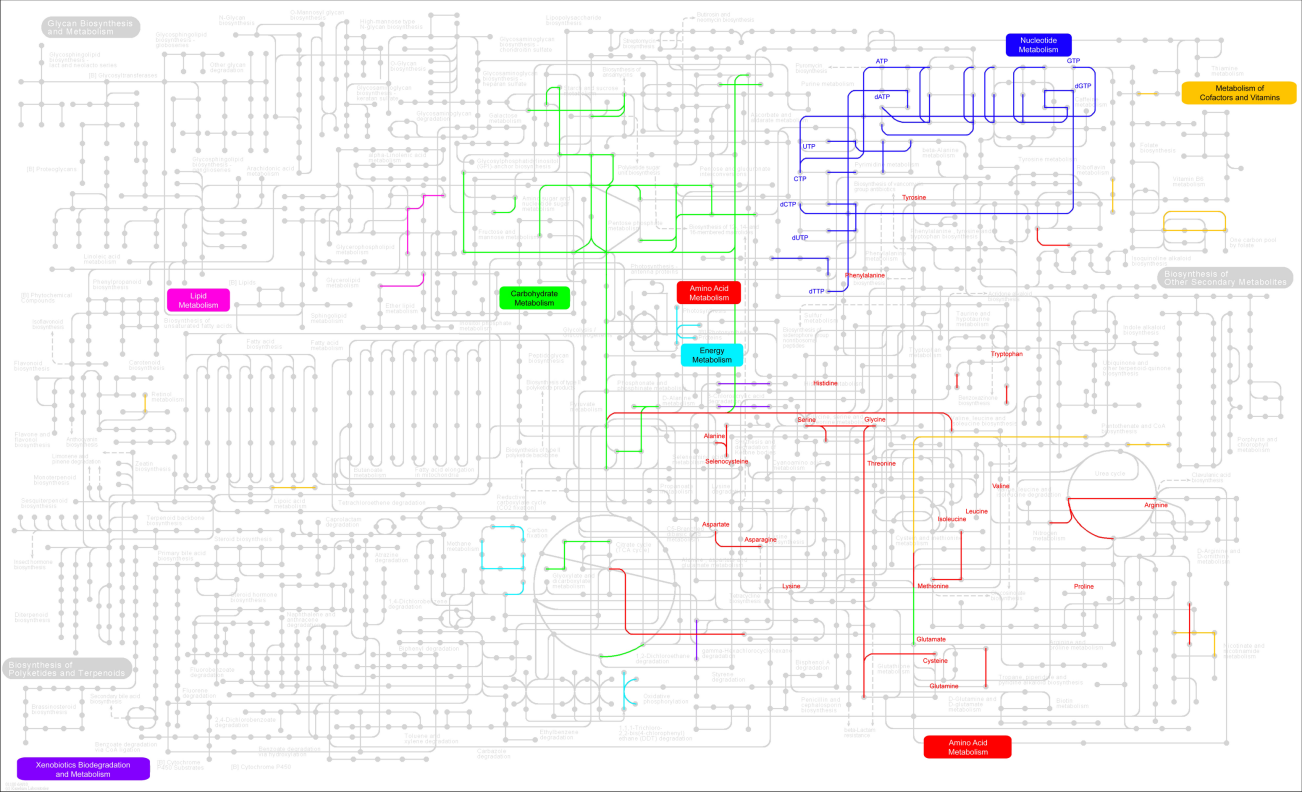

Supplement: Figure S4 — A global view of M. fermentans metabolic networks showing the connected reactions in carbohydrate (green), nucleotide (blue), amino acids (red), cofactors and vitamins (orange), and lipid (pink) metabolisms. The map is produced by matching the M64 predicted proteins to metabolic pathways with the “color pathway” tool in KEGG (http://www.genome.jp/kegg/tool/color_pathway.html). (TIF) [file pone.0032940.s004.tif]

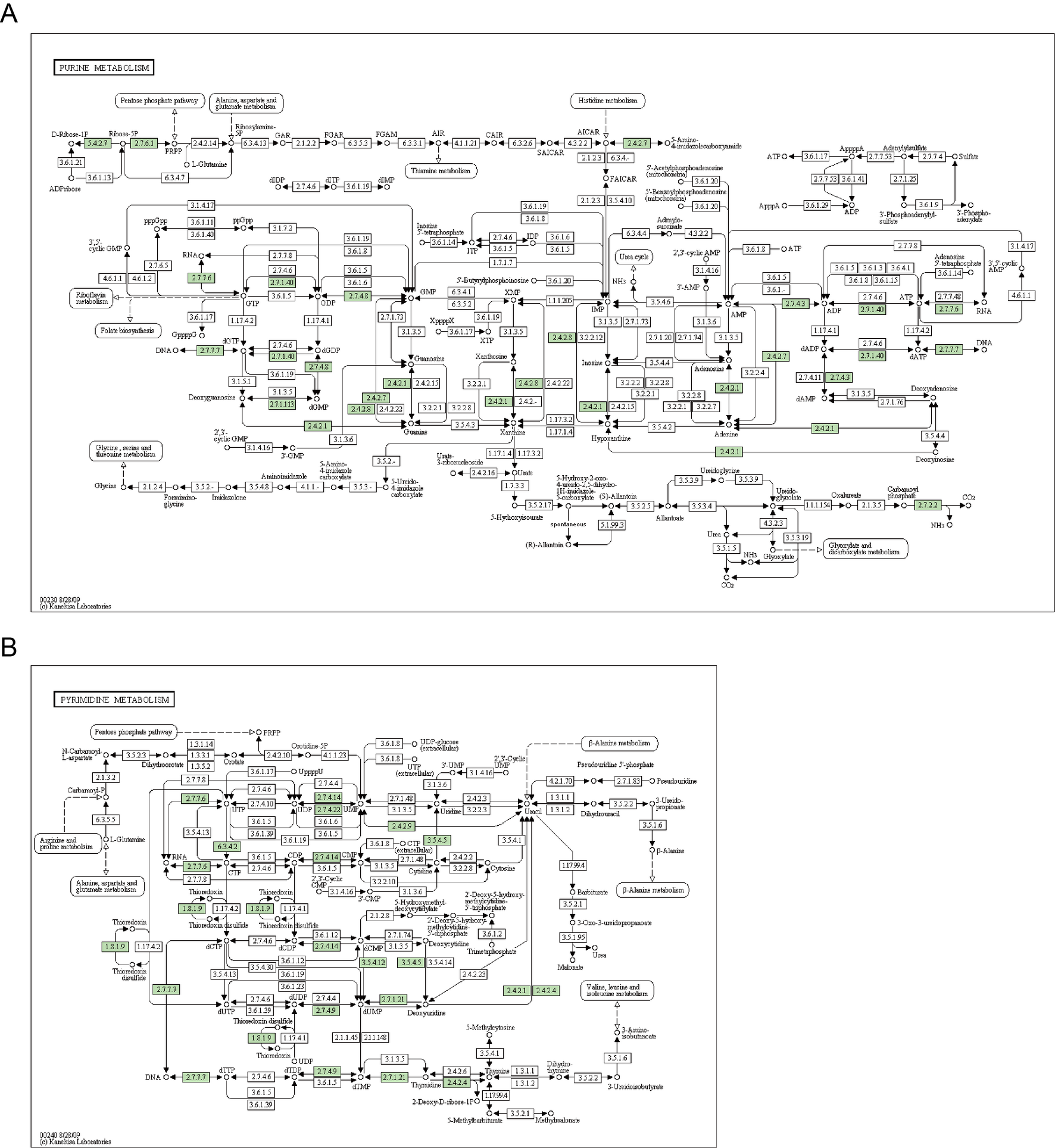

Supplement: Figure S5 — M. fermentans M64 purine (A) and pyrimidine (B) metabolic pathways contain many small clusters of connected reactions. Small circles represent the metabolic intermediates and green rectangles with enzyme catalog numbers denote the proteins in M. fermentans. (TIF) [file pone.0032940.s005.tif]

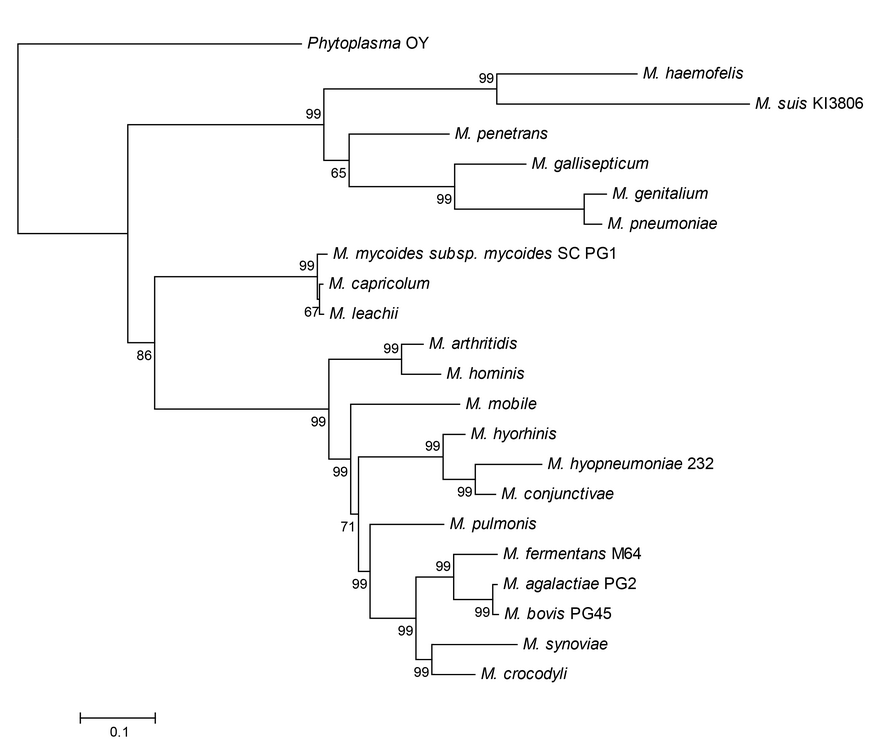

Supplement: Figure S6 — Phylogenetic tree of the 21 Mycoplasma species analyzed in Figure 4 . Maximum-likelihood method was performed on the 23S rRNA to reconstruct this tree using PHYML 3.0. The branch reliability was evaluated by implementing the aLRT method. Phytoplasma OY was set as the outgroup. The scale bar stands for the estimated number of nucleotide substitutions per site. (TIF) [file pone.0032940.s006.tif]

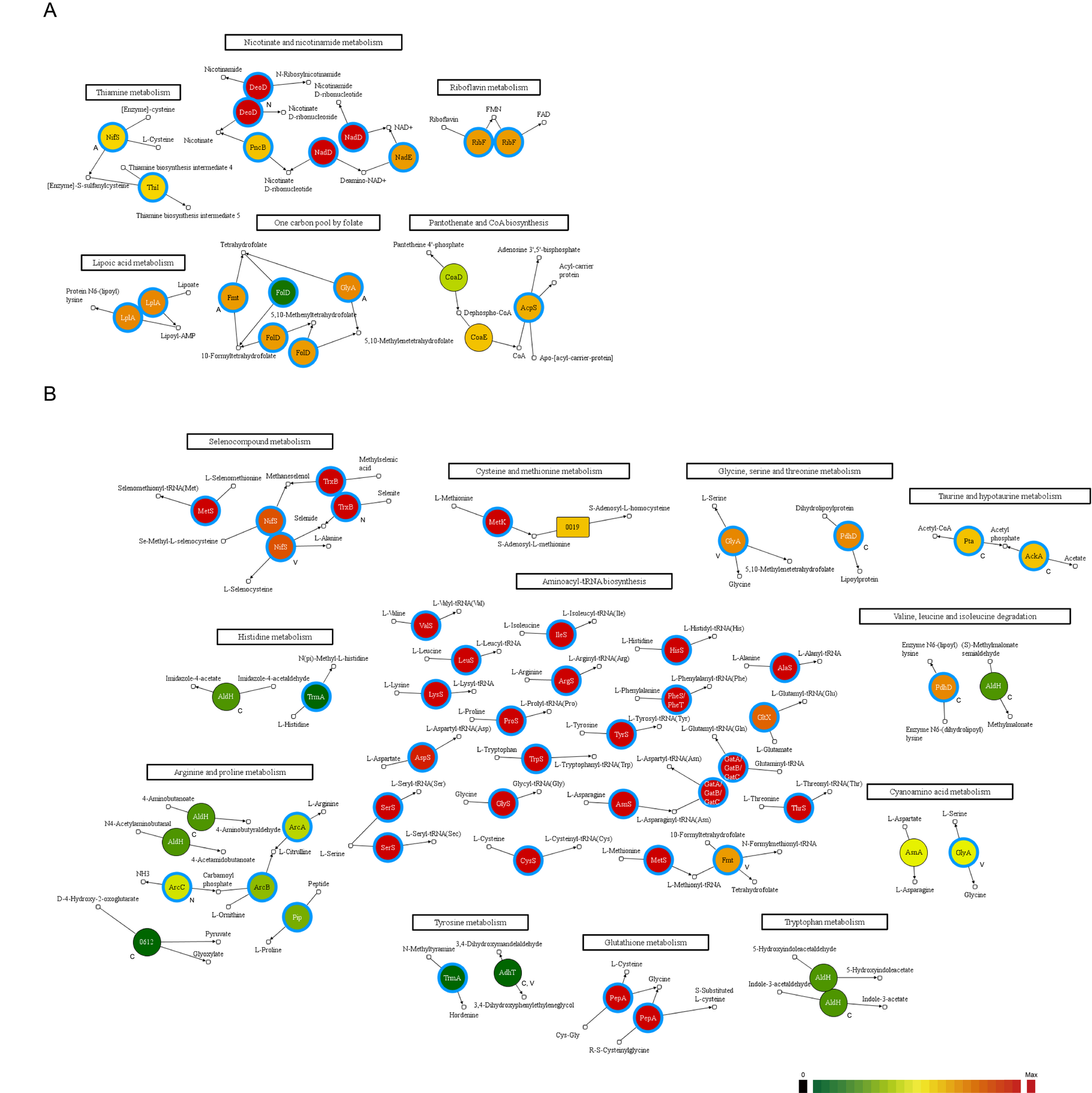

Supplement: Figure S7 — Phylogenetic comparative analysis of cofactors and vitamins (A) and amino acid (B) metabolic networks of M. fermentans M64 and 20 other Mycoplasma species. The small open squares represent the compounds and the colored circles represent the proteins (enzymes) participated in the metabolism. Circles with thick and light blue circumference represent the predicted essential genes. The arrows and lines linking the enzymes and substrates indicate the direction of the reactions. Small rectangle in B represents a putative horizontally transferred gene candidate. Enzymes involved in more than one metabolism are indicated with a one letter code (A: Amino acids metabolism; C: Carbohydrate metabolism; N: Nucleotide metabolism; V: metabolism and cofactors and Vitamins) next to the circles. The color scale (bottom) indicates the phylogenetic conservation of the proteins in 21 Mycoplasma species. (TIF) [file pone.0032940.s007.tif]

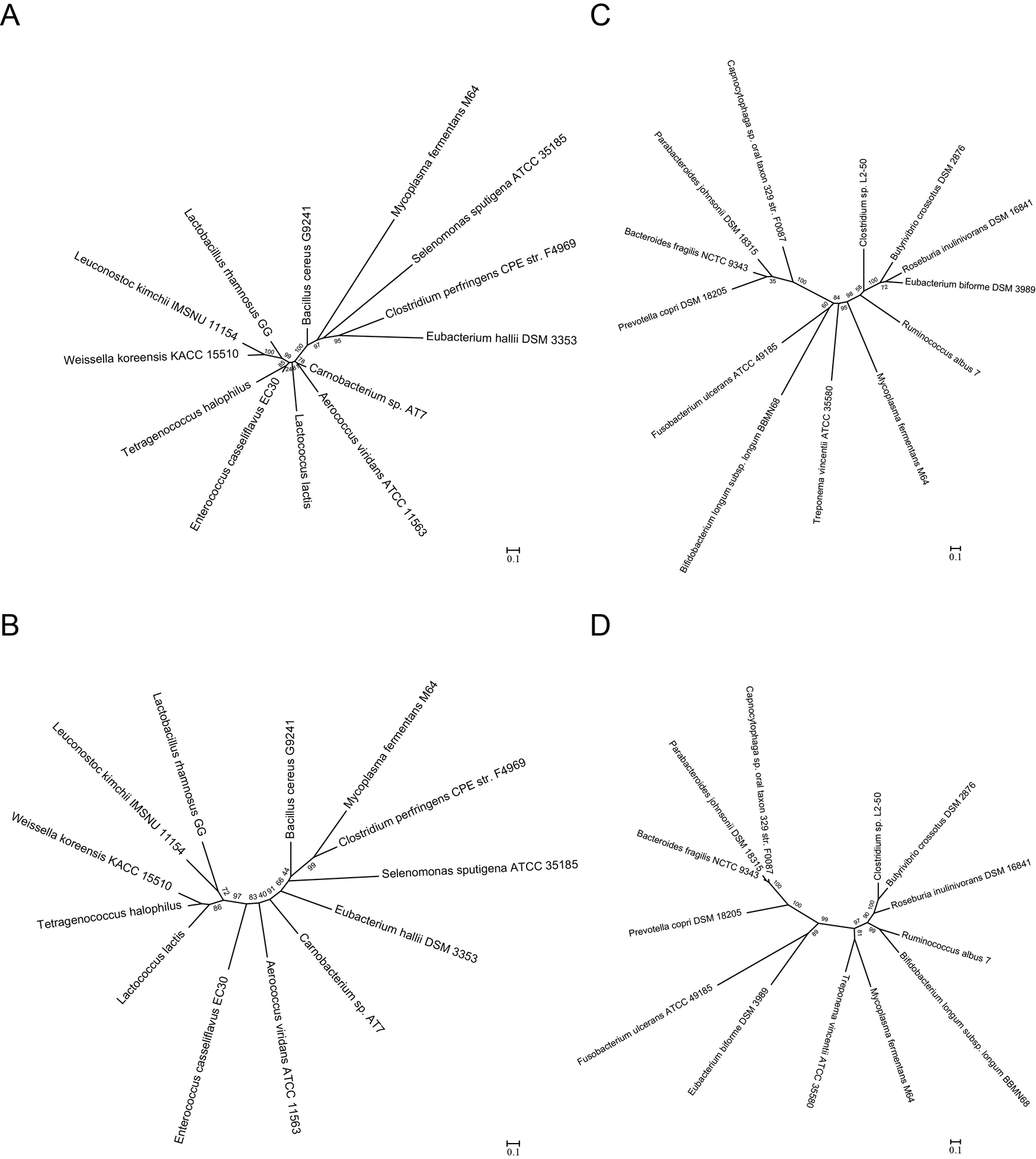

Supplement: Figure S8 — Phylogenetic relationships of MfeM64YM_1027 and MfeM64YM_0060 outside of the Mycoplasma clade. A. and C. The 23S rRNA trees of the species shown in B and D, respectively. B. and D. The phylogenetic trees of MfeM64YM_1027 and MfeM64YM_0060, respectively, and their closest homologs present in 12 other species. Maximum-likelihood method was used to reconstruct the trees. The branch reliability was evaluated by implementing the aLRT method. The scale bar stands for the estimated number of nucleotide (A and C) or amino acid (B and D) substitutions per site. (TIF) [file pone.0032940.s008.tif]
